# Murine Intraepithelial Dendritic Cells Interact With Phagocytic Cells During *Aspergillus fumigatus*-Induced Inflammation

**DOI:** 10.3389/fimmu.2020.00298

**Published:** 2020-02-25

**Authors:** Andrey O. Bogorodskiy, Elena L. Bolkhovitina, Thomas Gensch, Natalia I. Troyanova, Alexey V. Mishin, Ivan S. Okhrimenko, Armin Braun, Emma Spies, Valentin I. Gordeliy, Alexander M. Sapozhnikov, Valentin I. Borshchevskiy, Marina A. Shevchenko

**Affiliations:** ^1^Research Center for Molecular Mechanisms of Aging and Age-Related Diseases, Moscow Institute of Physics and Technology, Dolgoprudny, Russia; ^2^Laboratory of Cell Interactions, Department of Immunology, Shemyakin and Ovchinnikov Institute of Bioorganic Chemistry, Russian Academy of Sciences, Moscow, Russia; ^3^Institute of Biological Information Processing (IBI-1: Molecular and Cellular Physiology), Forschungszentrum Jülich, Jülich, Germany; ^4^Fraunhofer Institute for Toxicology and Experimental Medicine ITEM, Biomedical Research in Endstage and Obstructive Lung Disease Hannover (BREATH), Member of the German Center for Lung Research (DZL), Member of Fraunhofer International Consortium for Anti-Infective Research (iCAIR), Hanover, Germany; ^5^Institute of Immunology, Hannover Medical School, Hanover, Germany; ^6^Institute of Biological Information Processing (IBI-7: Structural Biochemistry), Forschungszentrum Jülich, Jülich, Germany; ^7^Institut de Biologie Structurale, Université Grenoble Alpes-CEA-CNRS, Grenoble, France

**Keywords:** intraepithelial dendritic cells, *Aspergillus fumigatus*, mouse model, conducting airway mucosa, whole-mount specimens, fluorescent confocal laser-scanning microscopy

## Abstract

People are constantly exposed to airborne fungal spores, including *Aspergillus fumigatus* conidia that can cause life-threatening conditions in immunocompromised patients or acute exacerbations in allergics. However, immunocompetent hosts do not exhibit mycoses or systemic inflammation, due to the sufficient but not excessive antifungal immune response that prevent fungal invasion. Intraepithelial dendritic cells (IE-DCs) of the conducting airway mucosa are located in the primary site of the inhalant pathogen entry; these cells can sense *A. fumigatus* conidia and maintain homeostasis. The mechanisms by which IE-DCs contribute to regulating the antifungal immune response and controlling conidia dissemination are not understood. To clarify the role of IE-DCs in the balance between pathogen sensing and immune tolerance we investigated the *A. fumigatus* conidia distribution in optically cleared mouse lungs and estimated the kinetics of the local phagocytic response during the course of inflammation. MHCII^+^ antigen-presenting cells, including IE-DCs, and CD11b^+^ phagocytes were identified by immunohistochemistry and three-dimensional fluorescence confocal laser-scanning microscopy of conducting airway whole-mounts. Application of *A. fumigatus* conidia increased the number of CD11b^+^ phagocytes in the conducting airway mucosa and induced the trafficking of these cells through the conducting airway wall to the luminal side of the epithelium. Some CD11b^+^ phagocytes internalized conidia in the conducting airway lumen. During the migration through the airway wall, CD11b^+^ phagocytes formed clusters. Permanently located in the airway wall IE-DCs contacted both single CD11b^+^ phagocytes and clusters. Based on the spatiotemporal characteristics of the interactions between IE-DCs and CD11b^+^ phagocytes, we provide a novel anatomical rationale for the contribution of IE-DCs to controlling the excessive phagocyte-mediated immune response rather than participating in pathogen uptake.

## Introduction

The drug resistance-mediated expansion of infections caused by fungi, including *Aspergillus fumigatus*, makes further investigation of antifungal immune response mechanisms of great importance (1, 2). Dendritic cells (DCs) contribute to defense against *A. fumigatus*-induced infections and are considered as potential target cells for the development of new immune augmentation therapeutic strategies (3, 4). In the airways, mucosal DCs form a network that permits the sensing of inhaled pathogens such as *A. fumigatus* conidia (5, 6). DCs of the airway mucosa function as professional antigen-presenting cells (APCs) and can sample airborne antigens and activate the adaptive immune response, either systemically in the draining lymph nodes or locally in the periphery (7, 8). Beyond activation of adaptive immunity, tissue-resident DCs control the local innate immune response that is crucial in acute inflammation (9–11). Airway DCs comprise three subsets: two conventional types (cDC1s and cDC2s) and plasmacytoid pDCs, which can be defined using a set of lineage-imprinted markers either in tissue homogenates by flow cytometry and mass cytometry (12) or in tissue specimens by an immunohistochemistry-based method (13). Notably, immunohistochemistry of thick tissue specimens permits the determination of the location and microenvironment of a certain cell, but the identification of DC subsets is restricted to the use of a limited number of simultaneously detected markers. Lung pDCs and cDCs have been discriminated using immunohistochemistry (13). However, no strong correspondence between cDC1/cDC2 and CD103^+^CD11b^−^/CD103^−^CD11b^+^ cDC subsets detected by immunohistochemistry was reported. At the same time, a significant reduction of lung CD103^+^ cDC numbers was detected in *Batf3*^−/−^ mice (14). Since cDC1 development was shown to be BATF3-dependent (12), this observation supports the evidence that detected by immunohistochemistry CD103^+^CD11b^−^cells can be classified as cDC1s. In the conducting airways under non-inflamed conditions, cDCs mostly express CD11c and MHCII, possess heterogeneous morphology and locate differently in relation to the epithelial and smooth muscle layers (15). It has been shown indirectly that CD103^+^CD11b^−^cDCs are characterized by irregular shapes with multiple dendrites, while CD103^−^CD11b^+^ cDCs possess amoeboid shapes (16). cDC subsets occupy distinct microanatomical compartments: CD103^+^CD11b^−^ cells are located within the epithelial barrier in close proximity to epithelial cells and above the basement membrane, here termed intraepithelial DCs (IE-DCs), while CD103^−^CD11b^+^ cells–beneath the smooth muscle layer in the submucosal compartment (5, 16). Conducting airway mucosal DCs possess distinct motility characteristics: IE-DCs are sessile with dendrites adhered to the epithelium, while amoeboid subepithelial DCs are motile and migrate randomly (5). Although IE-DCs are commonly represented by CD103^+^CD11b^−^ cDCs (16), the shape of these cells is dictated by their tissue location, by the need to squeeze through between the epithelial cells and to a lesser extent by their developmental origin (T.Z. Veres, personal communication, January 2018). Thus, a branched shape may potentially be attributed to different DC subsets.

The role of conducting airway mucosal DC subsets in the *A. fumigatus*-induced inflammatory response is still under investigation. cDC2s can potentially contribute to the direct internalization of conidia via CD11b and complement-dependent types of phagocytosis (17). DCs can also sense and internalize fungal spores via pattern recognition receptors such as the lectin receptors Dectin-1 and Dectin-2 and the mannose receptor DCSIGN, which act in cooperation with Toll-like receptors, mainly TLR2 and TLR4 (18). Previously, we have shown that intraepithelial MHCII^+^ cells with irregular shapes (intraepithelial APCs) occasionally ingested *A. fumigatus* conidia in the conducting airways of mice with preexisting allergic sensitization (6). The fact that IE-DCs are mainly represented by CD103^+^CD11b^−^ cDCs (16), which could be classified as cDC1s, allows to suppose IE-DCs to potentially contribute to the control of inflammation and the maintenance of homeostasis in the airways (9, 10).

People inhale approximately from hundred to thousand *A. fumigatus* conidia daily (1). However, inhalation of conidia does not cause fungal contamination or severe inflammation in immunocompetent hosts. Analysis of the spatiotemporal aspects of interactions between inhaled conidia and immune cells in the natural microenvironment would provide deep insight into the balance between pathogen sensing and immune tolerance.

Here, we focus on the local innate immune response in the conducting airways of mice that received a single dose of 5 × 10^6^ *A. fumigatus* conidia via the oropharyngeal route. To clarify the role of conducting airway IE-DCs in the antifungal response, we estimated the kinetics of APCs in conducting airway mucosa during the course of *A. fumigatus* conidia-induced inflammation. We characterized the inflammation-induced changes in intraepithelial APC subsets of the conducting airway wall: IE-DCs and CD11c^−^ APCs. We also investigated the ingestion effectiveness of CD11c^+^ cells and CD11b^+^ phagocytes in the conducting airway mucosa. Spatiotemporal analysis of the interactions between IE-DCs and CD11b^+^ phagocytes revealed the formation of direct contacts between IE-DCs and the swarming clusters of CD11b^+^ phagocytes that infiltrated the airway wall in response to *A. fumigatus* conidia. Our data provide evidence that such interactions can play important roles in the local control of the acute inflammation in the airways.

## Materials and Methods

### Animals and Ethics Statement

CD11c-EYFP mice on C57BL/6 background (19) were kindly gifted by Prof. Michel C. Nussenzweig (The Rockefeller University, New York, NY), bred in animal facility of Shemyakin and Ovchinnikov Institute of Bioorganic Chemistry, Russian Academy of Sciences; male (18–30 weeks old) were used in this study. All animal experiments were performed in concordance with the Guide for the Care and Use of Laboratory Animals under a protocol approved by the Institutional Animal Care and Use Committee at Shemyakin and Ovchinnikov Institute of Bioorganic Chemistry Russian Academy of Sciences (protocol number 245/2018). Animals were given standard food and tap water *ad libitum* and housed under regular 12-h dark: light cycles at 22°C.

### *Aspergillus fumigatus* Strain

The *A. fumigatus* strain AfS150 (20), a ATCC 46645 derivative constitutively expressing the dTomato fluorescent protein, was used in this study. Conidia of this isolate had been obtained from Prof. Sven Krappmann (University Hospital Erlangen and FAU Erlangen-Nürnberg, Germany).

Conidia were fixed overnight with 3% paraformaldehyde (Sigma–Aldrich, Seelze, Germany), washed twice with Dulbecco's Phosphate-Buffered Saline (DPBS) (PanEco, Moscow, Russia). Since the fluorescence of dTomato fluorescent protein was lost after fixation, conidia were labeled with Alexa Fluor 594 NHS Ester (Thermo Fisher, Eugene, OR) or Alexa Fluor 700 NHS Ester (Thermo Fisher) according to the manufacturer's instructions. Conidia were filtered through 10 μm Nylon Net Filter (Millipore, Cork, Ireland), aliquoted, and stored at 4°C until use.

### *A. fumigatus* Conidia Application

Paraformaldehyde fixed *A. fumigatus* conidia were dissolved in DPBS to a concentration of 1 × 10^8^ conidia/mL. Mice were anesthetized by inhalation of isoflurane (Baxter, Guayama, Puerto Rico) and a 50-μL droplet containing 5 × 10^6^ of conidia (unless otherwise indicated) was applied to the oropharyngeal cavity of each mouse (21).

### Neutrophil Depletion

Neutropenia was mimicked by injecting neutrophil-depleting antibodies rat anti-mouse Ly6G (BioLegend), clone 1A8, 170 μg per mouse. Antibody dosage was chosen in accordance with our previous findings (22). Control groups received rat IgG2a (BioLegend), 170 μg per mouse. All antibodies and isotype controls were diluted in DPBS to a total volume of 200 μl and administered via intraperitoneal injection 1 day prior to *A. fumigatus* conidia application.

### Whole-Mount Lung Lobe Specimen Preparation, Staining, and Optical Clearing

Animals were euthanized and their lungs were harvested and fixed without inflation with 2% paraformaldehyde within 2 h at room temperature. Lung lobes were initially washed with Tris-buffered saline (TBS), pH 7.4, 5 times each for 1 h and then blocked overnight with 0.3% Triton X-100 (Helicon, Moscow, Russia) and 5% powdered milk (Roth, Karlsruhe, Germany) in TBS at room temperature at 150 rpm on a shaker (Apexlab, Moscow, Russia). The airways were labeled with streptavidin conjugated to Alexa Fluor 488 (Thermo Fisher) for 3 days (23). Specimens were washed in TBS as it was mentioned above and postfixed overnight in 2% paraformaldehyde. Lung lobe optical clearing was performed at room temperature on a sample mixer MXIC1 (Thermo Fisher). For dehydration specimens were incubated with 50% methanol for 1 h and then with 100% methanol for 2 h. For clearing specimens were immersed in 1 mL of a mixture of one volume of benzyl alcohol and two volumes of benzyl benzoate (BABB) for at least 20 min. Then, the lung lobes were placed into cell imaging coverglass chambers (Eppendorf, Hamburg, Germany) and stored until the time of microscopy.

### Whole-Mount Conducting Airway Specimen Preparation and Staining

Lungs were inflation-fixed with 2% paraformaldehyde and stored at 4°C overnight. The main bronchi from lung lobes (left and right inferior) were dissected. The airways were then washed with DPBS, permeabilized with 0.3% Triton X-100, and blocked with 1% bovine serum albumin (Serva, Heidelberg, Germany) and 4% normal goat serum and/or normal donkey serum (Jackson Immuno Research, Cambridge, UK). The following antibodies and dilutions were used: purified anti-mouse I-A/I-E (BioLegend), 1:50; purified rat anti-mouse Ly6G/Ly6C (BioLegend), clone RB6-8C5, 1:50; Alexa Fluor594-conjugated anti-mouse Ly6G (BioLegend), clone 1A8, 1:50; purified rat anti-mouse CD11b (BioLegend), 1:50; allophycocyanin-conjugated anti-mouse CD11b (BioLegend), 1:50; Alexa 555-conjugated goat anti-rat IgG (Thermo Fisher), 1:250; Alexa 594-conjugated donkey anti-rat IgG (Thermo Fisher), 1:250. Phalloidin-Atto 490 LS (Sigma) and Hoechst 33342 (Thermo Fisher) were used according to manufacturers recommendation. All samples were mounted in Prolong Gold mounting medium (Thermo Fisher).

### Confocal Laser-Scanning Microscopy

An inverted confocal LSM780 microscope (Zeiss, Jena, Germany) was used in all experiments with either a 10× (NA = 0.3), 40× (NA = 1.4, water immersive), or a 100× (NA = 1.46, oil immersive) objective. Excitation at 405, 488, 561, and 633 nm was used to visualize Hoechst 33342, Alexa Fluor 488, Alexa Fluor 555/594, allophycocyanin and Alexa 700 fluorescence, respectively. Emission was measured in CLSM λ-mode using a 34-channel QUASAR detector (Zeiss) set to a 405–695 nm range. For quantitative analysis, images were captured as 2 × 2 tile grids at the same regions of each specimen using the 40 × objective, with an individual xyz tile size of 354 × 354 × 30 μm. Higher magnification images were acquired in z-stacks at the region of interest using the 100× objective. Spectral unmixing was performed using ZEN 2012 SP5 software (Zeiss). Finally, the images were processed using Adobe Photoshop CS version 5 (Adobe Systems, Mountain View, CA).

### Quantitative Image Analysis

Image stacks were analyzed using Imaris version 7.6.5 software (Bitplane Scientific Software, Zurich, Switzerland). MHCII^+^ APCs, CD11c^+^ cells, Ly6G^+^ neutrophils, CD11b^+^ phagocytes and *A. fumigatus* conidia, along with the epithelial and smooth muscle layers, were identified and processed via “three-dimensional surface rendering” of the appropriate channel, as previously described (22). The threshold and filter settings were optimized by visually comparing the result with the maximum intensity projection. Based on the epithelium and smooth muscle layer position, the “Crop 3D” function was applied to each image to obtain the appropriate region for quantification. The cell number was automatically calculated from the respective surface objects. Visual inspection was performed to confirm the accuracy of the automated quantitation results. The number of IE-DCs was quantified manually.

### Statistical Analysis

The data are presented as the graph or the scattered dot plot with the median and interquartile range (IQR) for at least 4 mice. Each point is an average of values that were obtained from different regions of the specimen (*n* = 2–4). The differences between two groups were analyzed with the Mann–Whitney *U* test using GraphPad Prism 7 software (GraphPad Software, San Diego, CA). A *p* < 0.05 was considered statistically significant.

## Results

### Conducting Airway Mucosal Antigen-Presenting Cell Response to *A. fumigatus* Conidia

**Figure 1:**
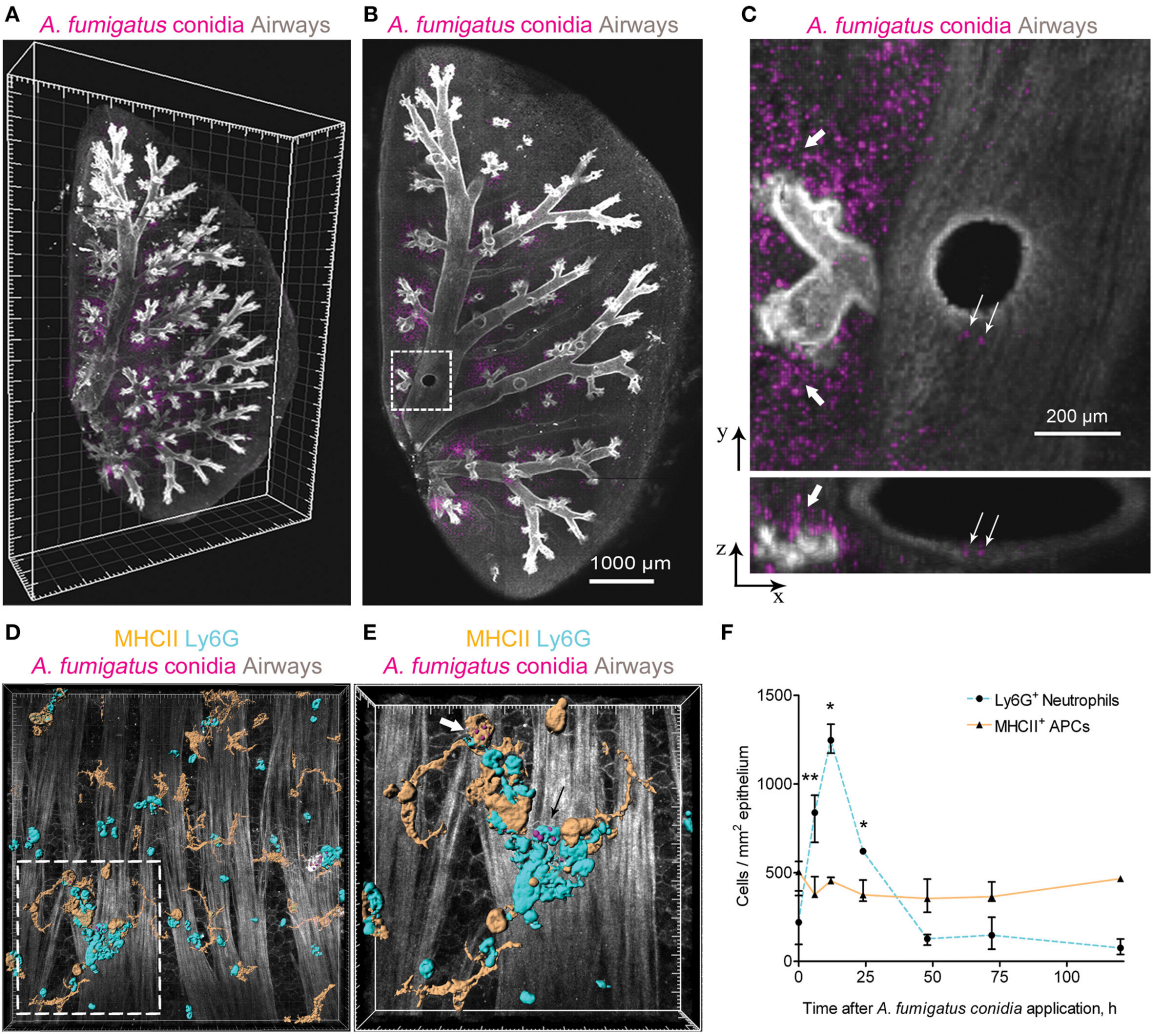
*A. fumigatus* conidia-mediated changes in intraepithelial APCs of the conducting airways. **(A)** Localization of *A. fumigatus* conidia in the bronchial compartment using optically cleared whole-lung specimens. Representative image of the optically cleared left lung lobe of mice obtained 72 h after 5 × 10^6^ *A. fumigatus* conidia application. Airways (gray) and conidia (magenta) are represented via volume rendering. Grid spacing, 500 μm. (**B)** Slice of the specimen shown in **(A)**. Scale bar, 1,000 μm. **(C)** Region indicated in **(B)** is represented as x-y (upper image) and x-z (lower image) projections showing conidia in the alveolar space (bold arrows) and in the bronchial compartment (fine arrows). Scale bar, 200 μm. **(D)** Potential phagocytic cells: APCs (MHCII^+^, orange) and neutrophils (Ly6G^+^, cyan) in the conducting airway mucosa of mice 72 h after 1.5 × 10^7^ *A. fumigatus* conidia (magenta) application. APCs, neutrophils and conidia are represented via surface rendering, smooth muscles and epithelium (gray) via volume rendering. Grid spacing 20 μm. **(E)** Enlarged region that is boxed in (**D)**. Bold arrow indicates APC (MHCII, orange) that internalized conidia (magenta) and fine arrow indicates neutrophil (Ly6G, cyan) that internalized conidia (magenta). APCs, neutrophils and conidia are represented via surface rendering. Grid spacing, 10 μm. **(F)** Quantitative analysis of neutrophil (dotted cyan line) and APC (solid orange line) numbers in the conducting airway mucosa of mice that received 5 × 10^6^ conidia at different time points after conidia application (*n* = 4–12 mice per time point; *n* = 2–6 tiles per mouse). The data are shown as the median and IQR. Statistical analyses were performed using the Mann–Whitney *U*-test. Significant differences between the indicated time point and the 0 h time point (before conidia application) are indicated: *p ≤ 0.05; **p ≤ 0.01; if not indicated, no significant difference.

We performed three-dimensional imaging of optically cleared whole-mount lung lobe specimens of mice preliminarily exposed to a single dose of 5 × 10^6^ *A. fumigatus* conidia oropharyngeally (Figures 1A,B). Specimens were fixed by a procedure without inflation of lungs with fixative to prevent wash out of conidia to the alveolar compartment. We found that after a single dose of 5 × 10^6^ *A. fumigatus* conidia application, conidia were accumulated in the alveolar space (Figures 1B,C, bold arrows) and in the bronchial branches to a lesser extent (Figure 1C, fine arrows). In the latter case, a precise analysis revealed that conidia were located in the bronchial lumen in close proximity to the airway wall (Figure 1C, lower image, fine arrows); and retained there up to 72 h after conidia application (Figures 1A–C).

Adhesion of conidia to the airway epithelium triggers the activation of immune cells (24). Previously we have shown that neutrophils infiltrated the conducting airway wall in response to *A. fumigatus* conidia application and participated in the antifungal defense by the direct conidia internalization (22). In the present study, we checked whether mucosal APCs could also internalize conidia in the conducting airways of immunocompetent mice after a single dose of *A. fumigatus* conidia oropharyngeal application. We dissected the main bronchus and performed immunohistochemistry of whole-mount airways, as described previously (15, 22). APCs were identified according to MHCII expression (Figure 1D, orange), and neutrophils were stained for Ly6G (Figure 1D, cyan). To distinguish the conducting airway mucosa from the submucosal compartment, smooth muscles were visualized by staining their prominent actin filaments with phalloidin (Figures 1D,E gray). We observed that both neutrophils (Figure 1E, fine arrow) and conducting airway mucosal APCs (Figure 1E, bold arrow) internalized *A. fumigatus* conidia.

We performed a quantitative analysis of both neutrophils (Ly6G^+^) and APCs (MHCII^+^) in the conducting airway mucosa at different time points after the oropharyngeal application of *A. fumigatus* conidia. Robust neutrophil recruitment to the airways resulted in a significant increase in neutrophil numbers at 6, 12 and 24 h after conidia application (Figure 1F, dashed line). At 48 h and later after conidia application the neutrophil number was not significantly different from that before conidia application. Meanwhile, the number of conducting airway mucosal APCs was maintained without significant alterations during the course of inflammation (Figure 1F, solid line).

Neutrophils infiltrated the conducting airway mucosa during the acute phase of *A. fumigatus* conidia-induced inflammation, while APCs were present in the airway mucosa continuously. Mucosal APCs of the conducting airways along with neutrophils can interact with *A. fumigatus* conidia to participate in the antifungal defense.

### Conducting Airway IE-DC Kinetics During the Course of *A. fumigatus* Conidia-Induced Inflammation

**Figure 2:**
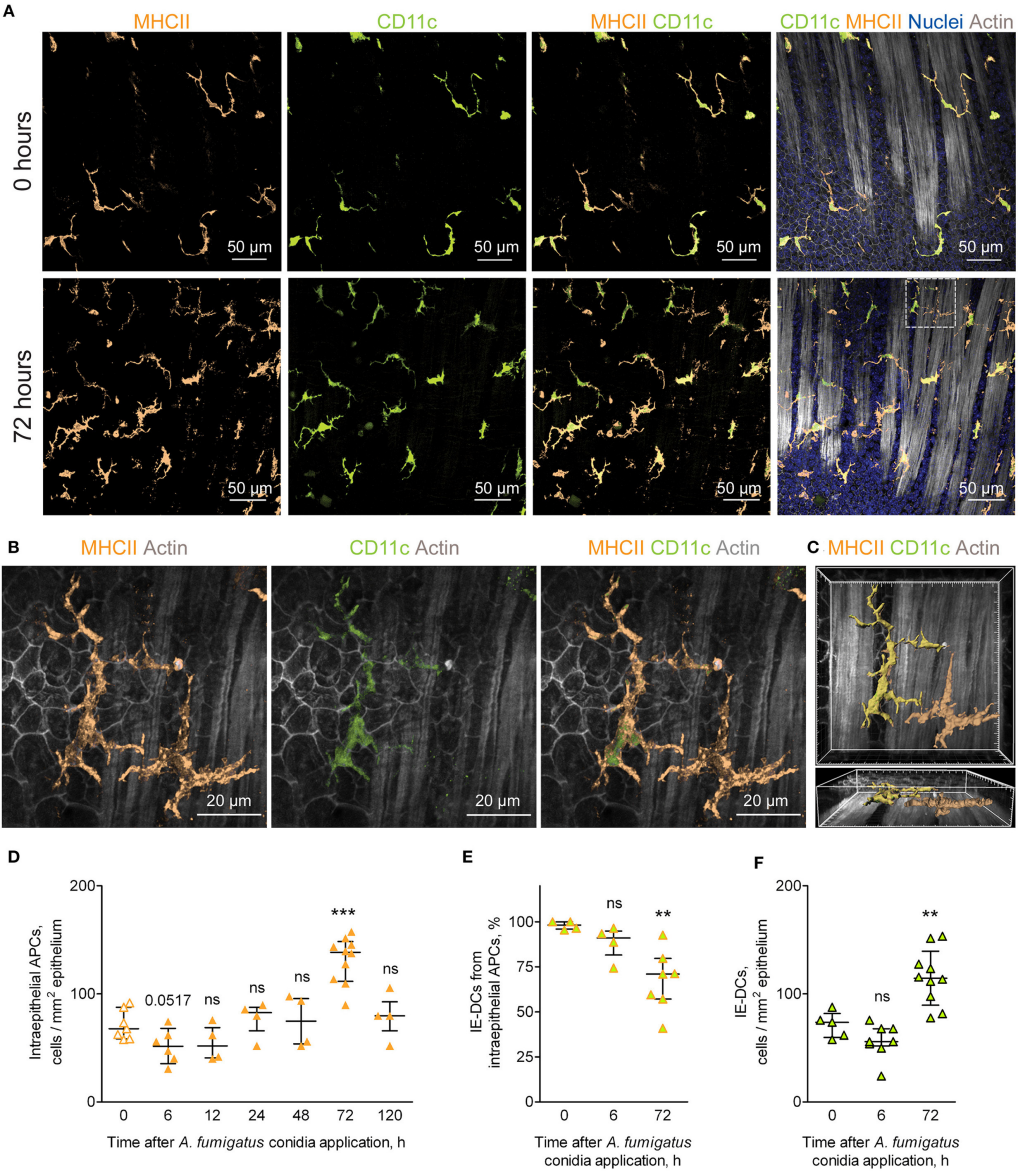
APCs of the conducting airway walls of uninfected and *A. fumigatus* conidia-infected mice. **(A)** Representative images of the conducting airway walls of uninfected mice (upper row) and of mice at 72 h after *A. fumigatus* conidia application (lower row). Intraepithelial APCs (MHCII, orange), including IE-DCs (CD11c, green), that are located between smooth muscles and the epithelium (Actin, gray) are represented via volume rendering. Cell nuclei are indicated in blue. Scale bar, 50 μm. **(B)** Higher magnification image of the region indicated in **(A)**, showing two APCs that are MHCII^+^ (orange), one of them is also CD11c^+^ (green) and therefore is related to IE-DCs. Scale bar, 20 μm. **(C)** Frontal (upper image) and lateral (lower image) three-dimensional images of the region indicated in **(A)** showing the precise position of IE-DC and CD11c^−^APC (MHCII^+^ CD11c^−^) in relation to the epithelium and smooth muscles (gray). APCs are represented via surface rendering, smooth muscles and epithelium via volume rendering. Grid spacing, 5 μm. **(D)** Quantitative analysis of the intraepithelial APC numbers at different time points after conidia application in immunocompetent mice (*n* = 4–10 mice per time point; each data point is an average *n* = 2–6 tiles per mouse). **(E)** The proportion of IE-DCs among intraepithelial APCs at 0, 6, and 72 h after conidia application (*n* = 4–7 mice per time point; each data point is an average *n* = 2–4 tiles per mouse). **(F)** The numbers of IE-DCs at 0, 6, and 72 h after conidia application (*n* = 5–10 mice per time point; each data point is an average of *n* = 2–4 tiles per mouse). The data are shown as the median and IQR. Statistical analyses were performed using the Mann–Whitney *U* test. Significant differences between the indicated time point and the 0 h time point (before conidia application) are indicated: **p ≤ 0.01; ***p ≤ 0.005; ns, no significant difference.

While in the alveolar space alveolar macrophages were shown to ingest conidia and provide sufficient antifungal defense (25, 26), monocyte-mediated immune response in the bronchial branches and the conducting airway is not characterized. In the present study, we focused on the resident conducting airway wall DCs that express CD11c and MHCII, located within the epithelium above the epithelial basement membrane and possessed irregular shape with dendrites (Supplementary Figure 1), termed here intraepithelial DCs (IE-DCs). We showed that IE-DCs mainly represented conducting airway wall APCs in uninfected mice (Figure 2A, upper images). However, in contrast to the uninfected state, *A. fumigatus* conidia application triggered the recruitment of CD11c^−^ APCs (MHCII^+^CD11c^−^ cells) with the similar to IE-DCs morphology to the airway wall (Figure 2A, lower images). CD11c^−^ APCs were not observed in the airway wall of uninfected mice, but were detected 6 h after *A. fumigatus* conidia application (Supplementary Figures 1A–D) and after, particularly 72 h after conidia application (Figure 2A, lower images, Figures 2B,C). Both IE-DCs and CD11c^−^ APCs were located in close proximity to the epithelial cells above the smooth muscles and above the epithelial basement membrane (Figure 2C and Supplementary Figure 1). Both IE-DCs and CD11c^−^ APCs formed the network of intraepithelial APCs in the conducting airway (Figures 2A,B).

The number of intraepithelial APCs accounted for one-fourth of the total number of conducting airway mucosal APCs (Figures 1F, 2D, orange solid line). Therefore, the recruitment of CD11c^−^ APCs to the airway wall resulted in the alteration of the number of intraepithelial APCs, but did not influence significantly the total number of mucosal APCs (Figures 1F, 2D, solid line). The number of intraepithelial APCs was significantly higher in the late phase of inflammation (72 h) than in the uninfected state (Figure 2D). Moreover, the number of CD11c^−^ APCs was significantly elevated at 72 h in the airway wall (Supplementary Figure 1E). Recruitment of CD11c^−^ APCs reflected the proportion of IE-DCs among intraepithelial APCs. The percentage of IE-DCs decreased with the progression of inflammation and reached a significantly lower level at 72 h (Figure 2E). At the same time, the elevation of intraepithelial APCs that was observed at 72 h after the conidia application (Figure 2D) did not result from the infiltration of CD11c^−^ APCs only. The number of IE-DCs also increased significantly at 72 h compared to that of uninfected mice (Figure 2F).

We identified inflammation-biased heterogeneity of intraepithelial APCs in the conducting airways and showed the infiltration of CD11c^−^ APCs and IE-DCs to the airway wall during the late phase of *A. fumigatus* conidia-induced inflammation.

### Contribution of IE-DCs to *A. fumigatus* Conidia Internalization

**Figure 3:**
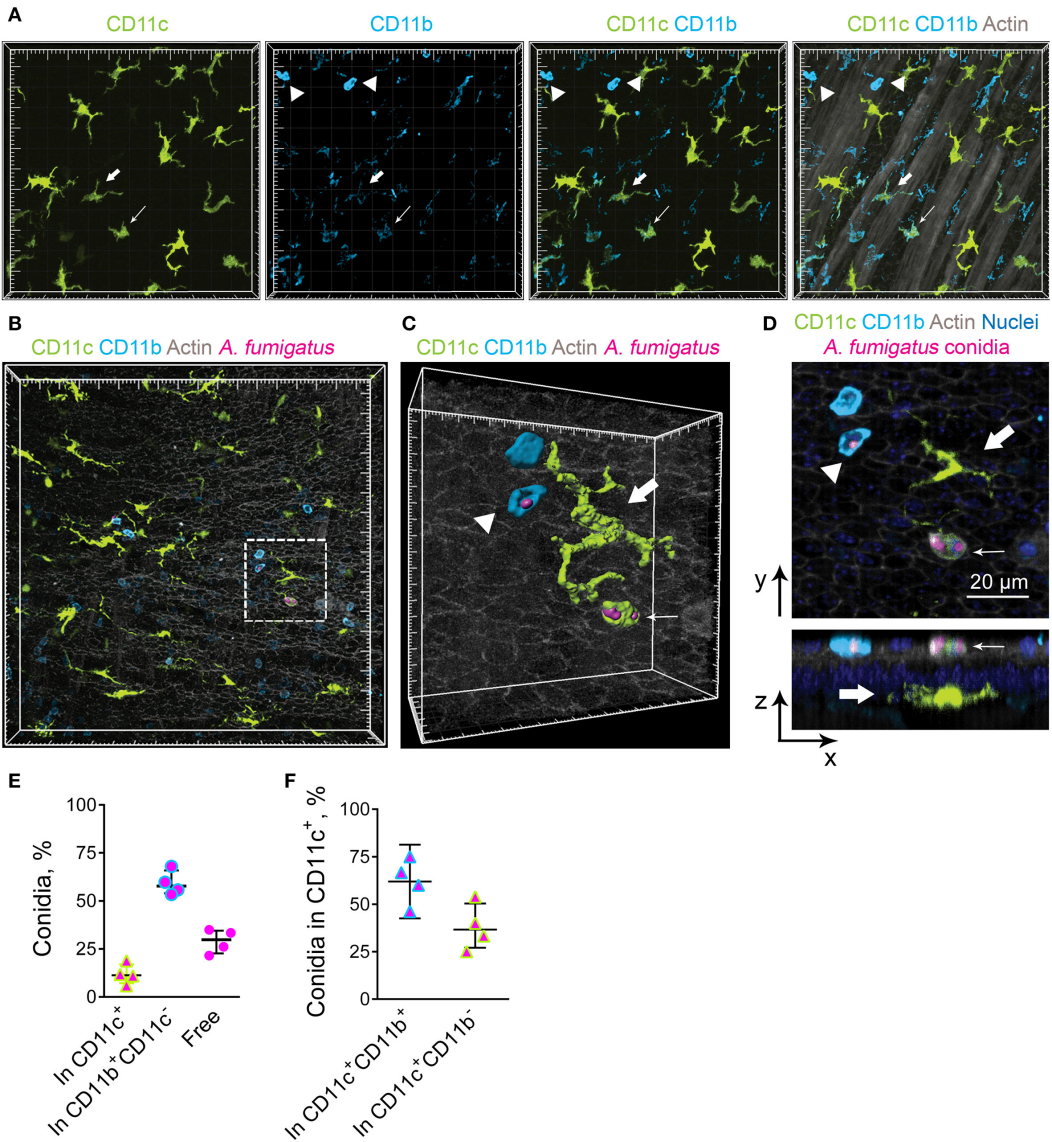
Airway mucosal CD11c^+^ cells and CD11b^+^ phagocytes contribute to conidia internalization. **(A)** Representative images of the conducting airway mucosa of mice 72 h after conidia application showing CD11c^+^ cells (green), particularly IE-DCs, expressing CD11b (light blue) (bold arrow), CD11c^+^ cells possessing an amoeboid shape and expressing CD11b (fine arrow) and CD11b^+^ phagocytes (CD11c^−^CD11b^+^, arrowheads). Images are shown as a volume rendering. Grid spacing, 20 μm. **(B)** Representative images of the events of *A. fumigatus* conidia (magenta) internalization by CD11c^+^ cell (green) or CD11b^+^ phagocyte (light blue) on the luminal side of the conducting airway epithelium (gray) 6 h after conidia application. Grid spacing, 20 μm. **(C,D)** Enlarged regions that are boxed in **(B)** showing the internalization of conidia (magenta) by CD11c^+^ cell (fine arrow) or by CD11b^+^ phagocyte (arrowhead). IE-DC is indicated by bold arrow. **(C)** Cells and conidia are represented via surface rendering, and the epithelium is represented via volume rendering. Grid spacing, 20 μm. **(D)** Frontal (upper image) and lateral (lower image) views of the region presented in **(C)** Scale bar, 20 μm. **(E)** Quantitative analysis of conidia that were internalized by CD11c^+^ cells (In CD11c^+^), CD11b^+^ phagocytes (In CD11b^+^CD11c^−^) and noninternalized 24 h after conidia application (*n* = 4 mice; at least 100 conidia per specimen). **(F)** Quantitative analysis of conidia that were internalized by CD11c^+^cells, either CD11c^+^CD11b^+^ cells (In CD11c^+^CD11b^+^) or CD11c^+^CD11b^−^ cells (In CD11c^+^CD11b^−^) 24 h after conidia application (*n* = 4 mice; at least 100 conidia per specimen). The data are shown as the median and IQR.

Previously we have observed occasional internalization of *A. fumigatus* conidia by intraepithelial APCs in mice with induced allergic sensitization (6). The aim of the present study was to detect whether conducting airway IE-DCs contribute to conidia internalization. *A. fumigatus* conidia are mainly ingested by complement-dependent phagocytosis (17). Complement receptors CR3 (CD11c/CD18) and CR4 (CD11b/CD18) are involved in opsonized pathogen internalization and digestion (27). As all DCs express CD11c and cDC2s express CD11b, which are components of CR3 and CR4, respectively, these cells possess phagocytic potential. To check the hypothesis, first, we examined for CD11b expression by the conducting airway IE-DCs. We observed that CD11b was prominently expressed by round-shaped cells that infiltrated the conducting airway wall or were identified at the luminal side of the epithelium (Figure 3A, arrowheads). Precise analysis revealed that these cells were mainly represented by Ly6G^+^ neutrophils, however, in some cases CD11b^+^ cells were Ly6G^−^ (Supplementary Figure 2). We also found several CD11c^+^CD11b^+^ cells with both amoeboid shape (Figure 3A, fine arrow) and dendrites (Figure 3A, bold arrow) in the airway wall. According to their location and morphology, the latter can be classified as IE-DCs. Notably, the expression of CD11b by conducting airway wall CD11c^+^ cells (Figure 3A, fine and bold arrows) was weaker than that by neutrophils (Figure 3A, arrowheads).

We next investigated the contribution of IE-DCs (either CD11b^+^ or CD11b^−^) and CD11b^+^CD11c^−^ cells, referred to hereafter as CD11b^+^ phagocytes, to *A. fumigatus* conidia uptake. We did not observe any IE-DC-conidia interactions. The cells that ingested conidia, both CD11c^+^ cells and CD11b^+^ phagocytes, were located on the luminal side of the conducting airway epithelium (Figures 3B–D, Supplementary Figure 3). Some IE-DCs were observed in close proximity to conidia (Figure 3B, Supplementary Figure 3), but three-dimensional imaging revealed the absence of contacts between IE-DCs and conidia (Figures 3C,D, Supplementary Figure 3). All internalizing conidia CD11c^+^ cells that were observed in the present study possessed a round shape and were located on the luminal side rather than IE-DCs that resided on the abluminal side of the epithelium (Figure 3D).

To estimate the contribution of conducting airway mucosal CD11c^+^ cells to conidia internalization, we quantified the number of CD11c^+^ cells (either CD11b^+^ or CD11b^−^) that internalized *A. fumigatus* conidia and compared it to that of CD11b^+^ phagocytes. During the acute phase of inflammation (from 6 to 24 h), the ingestion effectiveness of CD11b^+^phagocytes was higher than that of CD11c^+^ cells (Figure 3E). The number of conidia that were internalized by CD11b^+^ phagocytes (Figure 3E, In CD11b^+^CD11c^−^) was approximately three-fold higher than the number of conidia internalized by CD11c^+^ cells (Figure 3E, In CD11c^+^). Among ingesting CD11c^+^ cells, the percentage of conidia that were internalized by CD11c^+^CD11b^+^ cells exceeded 50% (Figure 3F).

Airway mucosal CD11c^+^ cells contributed substantially to the uptake of conidia of *A. fumigatus* in the conducting airways; however, the ingestion effectiveness of CD11b^+^ phagocytes (mostly neutrophils) was higher than that of CD11c^+^ cells. At the same time, IE-DCs that laid within the epithelium, but closer to the basement membrane than to the luminal side of the epithelial cells were unlikely to internalize conidia during the course of single-dose conidia-induced inflammation due to their spatial separation.

### CD11b^+^ Phagocytes Migrate Through the Conducting Airway Wall in Response to *A. fumigatus* Conidia Application

**Figure 4:**
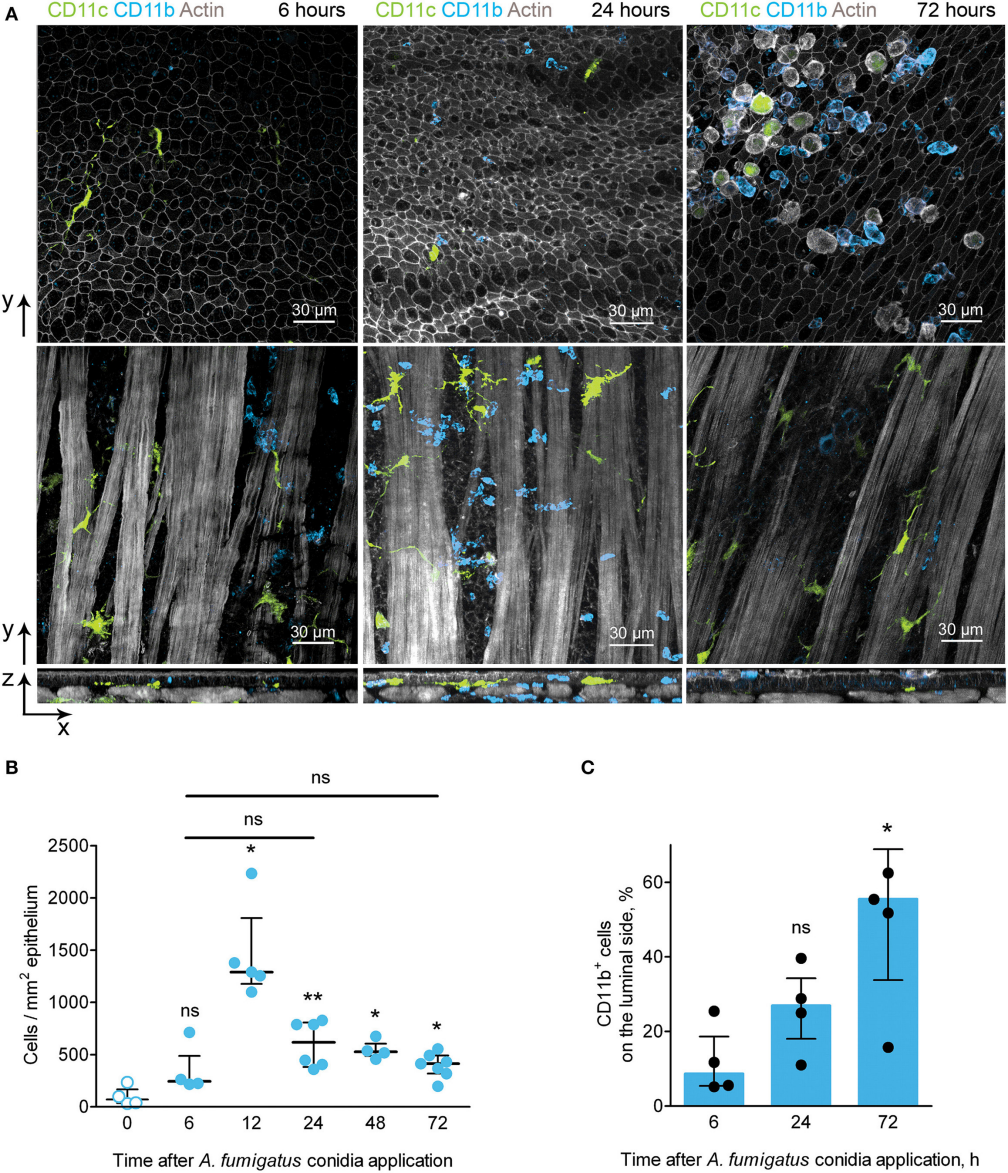
CD11b^+^ phagocyte kinetics and location during the course of *A. fumigatus* conidia-induced inflammation. **(A)** Representative images of CD11c^+^ cells (green) and CD11b^+^ phagocytes (light blue) of the conducting airway mucosa of mice at 6 (left images), 24 (middle images) and 72 (right images) h after conidia application. Cells and conidia are represented via volume rendering at frontal projections of the luminal side of the epithelium (upper images) and at frontal projections of the levels of smooth muscles (middle images) and at the lateral projections (lower images). Scale bar 30 μm. **(B)** Kinetics of conducting airway mucosal CD11b^+^ phagocytes in response to *A. fumigatus* conidia application (*n* = 4–7 mice per time point; each data point is an average *n* = 2–4 tiles per mouse). **(C)** Quantitative analysis of CD11b^+^ phagocytes on the luminal side of the conducting airway epithelial barrier at 6, 24, and 72 h after *A. fumigatus* conidia application (*n* = 4 mice per time point; each data point is an average *n* = 2–4 tiles per mouse). The data are shown as the median and IQR. Statistical analyses were performed using the Mann–Whitney *U*-test. The difference **(B)** between the indicated time point and the time point before conidia application (no conidia at 0 h) **(C)** between the indicated time point and the time point 6 h after conidia application is indicated: *p ≤ 0.05; **p ≤ 0.01; ns, not significant.

In the conducting airways *A. fumigatus* conidia (both free and internalized by immune cells) were observed mostly on the luminal side of the epithelium (Figures 3C,D). To be able to interact with conidia phagocytic cells must pass through the airway wall and reach the luminal side of the epithelium. Because of CD11b^+^ phagocytes were contributed the most to conidia uptake (Figure 3E), we identified the location of CD11b^+^ phagocytes during the course of *A. fumigatus* conidia-induced inflammation. Opposite to IE-DCs that were continuously detected in the airway wall (Figures 2D, 4A), CD11b^+^ phagocytes recruited to the airway wall in inflammation phase-dependent manner. At 6 h, CD11b^+^ phagocytes were already in the airway mucosa, but they were between smooth muscle fibers and in close proximity to the smooth muscle layer (Figure 4A, left images). At 24 h, the majority of these cells moved in close proximity to the epithelium (Figure 4A, middle images). CD11b^+^ phagocytes left the airway wall at 72 h after conidia application and were mostly located on the luminal side of the epithelium (Figure 4A, right images). Although some CD11c^+^ cells were identified at the luminal side of the epithelium, the number of IE-DCs did not decrease but instead increased significantly at 72 h after conidia application (Figure 2F). The observation supported the evidence that IE-DCs do not migrate from the airway wall to the luminal side of the epithelium. While IE-DCs were unlikely to migrate from the airway wall to the lumen, such migration was demonstrated for conducting airway mucosal CD11b^+^ phagocytes (Figure 4A).

We then quantified the number of CD11b^+^ phagocytes at different time points after conidia application and found that the number of these cells significantly increased at 12 h and remained increased at later time points up to 72 h after conidia application (Figure 4B). Similar kinetics were demonstrated above for conducting airway mucosal neutrophils (Figure 1F), that is in line with the observation that CD11b^+^ phagocytes of conducting airway mucosa were mainly represented by neutrophils (Supplementary Figure 2). Notably, there were no significant changes in the numbers of conducting airway mucosal CD11b^+^ phagocytes at 6, 24 and 72 h (Figure 4B). Quantitative analysis of the CD11b^+^ phagocyte migration showed a significant increase in these cell numbers on the luminal side of the epithelium at 72 h compared to that at 6 h after conidia application (Figure 4C).

Upon development of an inflammatory immune response, CD11b^+^ phagocytes migrated from the submucosa to the site of interaction with the pathogen–the luminal side of the conducting airway epithelium.

### IE-DCs Interact With Single CD11b^+^ Phagocytes and Swarming Phagocyte Clusters

**Figure 5:**
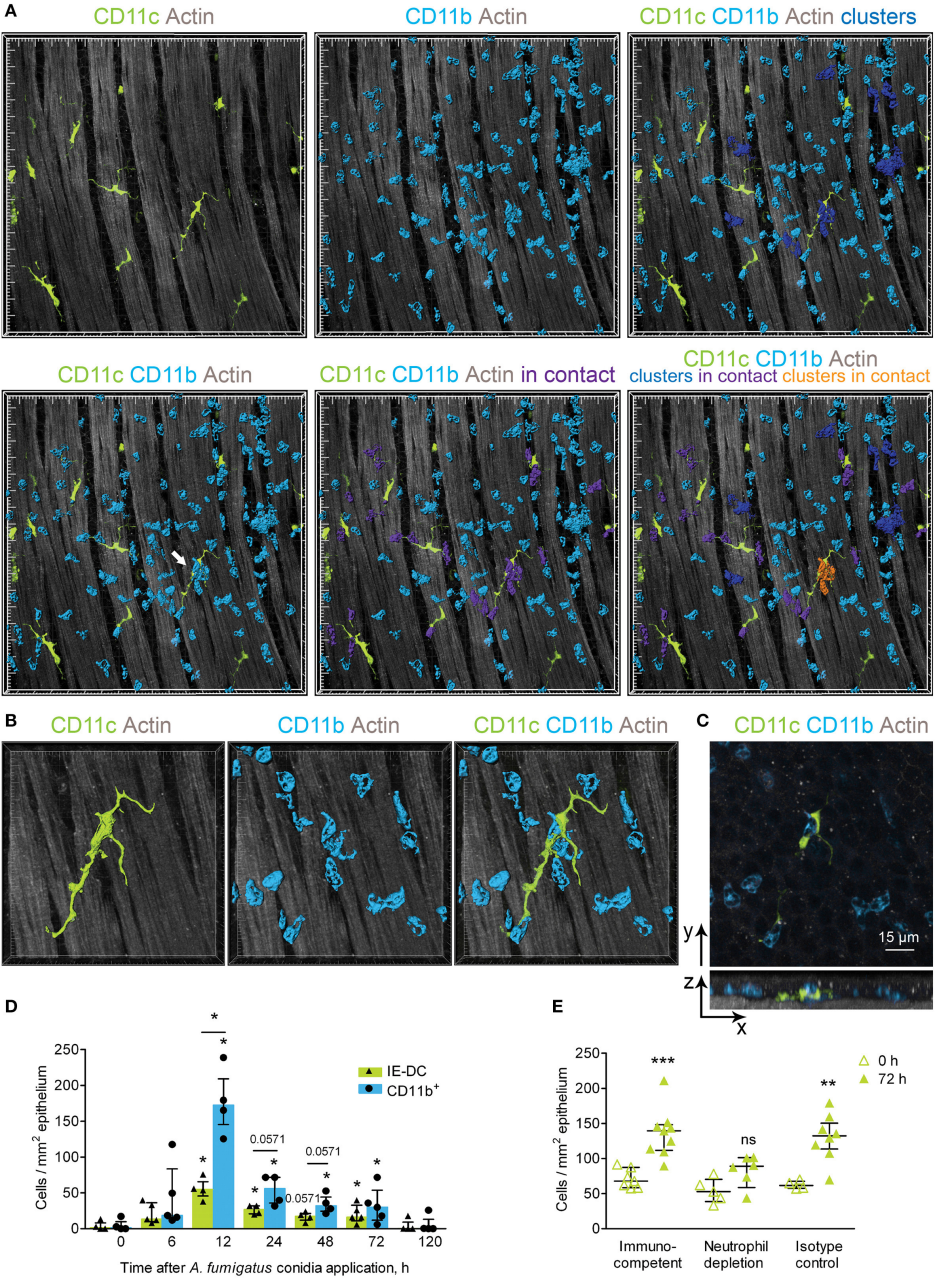
Implication of IE-DCs in the regulation of CD11b^+^ phagocyte swarming. **(A)** Representative images of a region of the conducting airway mucosa 12 h after *A. fumigatus* conidia application showing IE-DCs (green) and infiltrating conducting airway wall CD11b^+^ phagocytes (light blue). CD11b^+^ phagocytes are represented via surface rendering. IE-DCs (green), smooth muscles (gray) and the epithelium (gray) are represented via volume rendering. CD11b^+^ phagocytes that form clusters (more than two cells in close proximity) are indicated in blue (upper right image). CD11b^+^ phagocytes that are in contact with IE-DCs are indicated in violet (lower middle image). CD11b^+^ phagocyte clusters in contact with the IE-DC are indicated in orange (lower right image). Grid spacing, 20 μm. **(B)** Enlarged image of the IE-DC indicated in **(A)** showing a cluster of CD11b^+^ phagocytes (light blue) around the IE-DC (green). CD11b^+^ phagocytes and the IE-DC are represented via surface rendering, and the smooth muscle layer is represented via volume rendering. Grid spacing, 10 μm. **(C)** The image, represented in **(B)** as 3D, is presented as frontal (upper image) and lateral (lower image) projections. Cells are represented via volume rendering. Scale bar, 15 μm. **(D)** The numbers of interacting IE-DCs (green bars, triangles) and CD11b^+^ phagocytes (light blue bars, circles) at different time points after conidia application (*n* = 4–6 mice per time point; each data point is an average *n* = 2–4 tiles per mouse). **(E)** Quantitative analysis of the number of IE-DCs in the conducting airway of uninfected mice (open triangles) and at 72 h after *A. fumigatus* conidia application (green triangles). Data are shown for immunocompetent mice (Immunocompetent), mice that received rat anti-mouse Ly6G (Neutrophil depletion) and mice that received rat IgG2a (Isotype control) (*n* = 4–9 mice per time point; each data point is an average *n* = 2–6 tiles per mouse). The data are shown as the median and IQR. Statistical analyses were performed using the Mann–Whitney *U* test. **(D)** For IE-DCs and for neutrophils, the difference between the numbers at the indicated time point and the time point before conidia application (0 h) is indicated: *p ≤ 0.05; if not indicated, not significant. The difference between the neutrophil and IE-DC numbers interacting at indicated time points: *p ≤ 0.05; if not indicated, not significant. **(E)** The difference between the numbers at 0 and at 72 h is indicated: **p ≤ 0.01; ***p ≤ 0.005; ns, not significant.

CD11b^+^ phagocytes infiltrated the conducting airways upon inflammation and formed clusters in the conducting airway wall (Figure 5A, blue). Such swarming is well-established for neutrophils in response to pathogens or sterile tissue damage (28). Infiltrating conducting airway wall CD11b^+^ phagocytes interacted with IE-DCs (Figure 5A, violet). Notably, IE-DCs contacted with both single CD11b^+^ phagocytes (Figure 5A, lower right image, violet) and with phagocyte clusters (Figure 5A, lower right image, orange). Precise analysis of tight contacts revealed the formation of a structure resembling a phagocytic cap by IE-DCs around the neutrophil (Figures 5B,C). Our findings show that upon inflammation, CD11b^+^ phagocytes migrate to the conducting airway wall, where IE-DCs interact with both single phagocytes and with phagocyte clusters.

To clarify the role of IE-DC–CD11c^+^ phagocyte contacts in the course of *A. fumigatus* conidia-induced inflammatory response, we performed quantitative analyses of the interactions. In the conducting airway wall the swarming was the most pronounced at 12 h after conidia application, when the number of CD11b^+^ phagocytes was significantly increased (Figure 4B). The number of IE-DCs in contact with CD11b^+^ phagocytes increased significantly at 12, 24, and 72 h after conidia application compare to uninfected state (Figure 5D, green bars). At 48 h, this number was also increased, but not significantly (Figure 5D, green bars), while it returned to the level that was observed in uninfected mice by day 5 (120 h) after *A. fumigatus* conidia application (Figure 5D, green bars). Similarly, the number of CD11b^+^ phagocytes in contact with IE-DCs significantly increased at 12, 24, 48, and 72 h after conidia application in comparison to uninfected mice (Figure 5D, light blue bars). The number of interacting CD11b^+^ phagocytes at 12 h after application significantly exceeded the number of interacting IE-DCs, indicating that one IE-DC was in contact with more than one CD11b^+^ phagocyte (Figure 5D).

These contacts were detected up to 72 h after conidia application, but at 72 h, the CD11b^+^ phagocyte:IE-DC ratio in such contacts decreased (Figure 5D), in part due to a significant increase in IE-DC numbers (Figures 2D,F). Interestingly, in the case of neutrophil depletion by injection of depleting antibodies, no significant elevation of IE-DC number was detected at 72 h after conidia application (Figure 5E).

During the migration through the airway wall, CD11b^+^ phagocytes interacted with IE-DCs. We have shown here that the elevation of CD11b^+^ phagocyte (mostly neutrophil) numbers in the airway wall during the acute phase caused the upregulation of IE-DC numbers during the late phase of *A. fumigatus* conidia-induced inflammation.

## Discussion

It was previously accepted that *A. fumigatus* conidia penetrated the alveolar space due to their small size (2–3 μm) (1, 29). Alveolar macrophages were shown to ingest them and provide sufficient antifungal defense. Internalization of conidia by alveolar macrophages promotes conidia swollen and conidial pathogen-associated molecular patterns unmasking that facilitates conidia killing. However, dormant conidia can avoid the immune system recognition (25, 26). To reach the alveoli, conidia have to pass the bronchial branches and can retain there for some time (24). Thus, dormant conidia outside the alveolar space possess the fungal infection hazard. Here, using optically cleared whole-mount lung specimens, we observed fixed conidia in the alveolar space, but also in the conducting airways and bronchial branches as a result of the application of a single dose of 5 × 10^6^ *A. fumigatus* conidia. While alveolar macrophages do not reside in the conducting airways, investigating the role of resident monocytes in the innate immune response to conidia is of great importance.

In the present study, we investigated the role of IE-DCs in the conducting airways during the course of the immune response to a single oropharyngeal application of *A. fumigatus* conidia. We observed no evidence for IE-DCs involvement in pathogen uptake. IE-DCs did not contact conidia, but by the direct contact, they sensed neutrophil infiltration to the airway wall.

We show that the uptake efficiency of *A. fumigatus* conidia by conducting airway CD11c^+^ cells is location-dependent–it takes place on the luminal side of the airway epithelium and is not restricted to CD11c^+^CD11b^+^ cells only. CD11c^+^CD11b^−^ cells also contributed to conidia internalization, however, to a lesser extent. As a part of the MAC-1 complex, CD11b can be considered as an indicator of the ingestion effectiveness of the cell (30, 31). Recently, the importance of CD11b^+^ cells in defense against invasive aspergillosis was emphasized by the protective effect observed after the transfer of myeloid bone marrow-derived CD11b^+^ cells to infected mice with immunosuppression (32). In agreement with this previous finding, we have shown here that the ingestion effectiveness of CD11b^+^ phagocytes in the conducting airway mucosa was more than 50%, while CD11c^+^ cells ingested <25% of conidia. Predominantly, high expression of CD11b was attributed to conducting airway neutrophils; however, CD11c^+^CD11b^+^ cells were also occasionally found in the conducting airway wall. Besides, we observed CD11b^+^CD11c^−^Ly6G^−^ cells in conducting airway mucosa during the course of inflammation. These cells can belong to NK cells, which contribute to defense against pathogens, including *A. fumigatus* (33); however, this hypothesis should be further investigated. Our data indicate that not only CD11c^+^CD11b^+^ but also CD11c^+^CD11b^−^ cells interacted with conidia in the conducting airway lumen. The observation is in line with a report of Jakubzick et al. (7), who showed the involvement of CD11b^−^CD103^+^ pulmonary DCs in the corpuscular antigen uptake. Thus, although conducting airway mucosal CD11c^+^ cells are not the main contributors to fungal spore capture, they participate in conidia internalization. CD11c^+^ cells of the conducting airway mucosa obviously can implement not only CD11b^+^-mediated mechanism of conidia uptake, that is common for neutrophils (17), but also CD11c-mediated (27) or pattern-recognition receptor-mediated (18) internalization of *A. fumigatus* conidia.

Notably, the events of uptake were mostly observed on the luminal side of the conducting airway epithelium, and IE-DCs were separated from the conidia by the epithelial cells. Some CD11c^+^ cells that ingested conidia were found on the luminal side of the conducting airway epithelium; however, we do not expect them to be representatives of IE-DCs. Due to their branched shape and low motility (5), IE-DCs are not likely to squeeze through the epithelium to the luminal side to interact with conidia. The migration characteristics of these cells (34) also provide only weak evidence that intraepithelial DCs can egress from the airway wall to the airway lumen. Moreover, recent studies support the hypothesis that IE-DCs do not project their dendrites through the epithelial barrier to the airway lumen (5, 35). In agreement with these observations, here in the model of single-dose *A. fumigatus* conidia-induced inflammation we did not detect IE-DC–conidia interactions and concluded that IE-DCs were unlikely to catch and internalize fungal spores. In our previous study, we demonstrated that conidia uptake by intraepithelial APCs occurred in the case of preexisting sensitization to the allergen (6) but was not very frequent. It is also known that a layer of hydrophobic rodlet proteins that cover the dormant conidia make them inert, and degradation of these layers upon conidia germination is necessary for recognition and killing by macrophages and DCs (25, 26). In the present study, to exclude excessive immune system activation, we used paraformaldehyde-fixed conidia that mimicked the dormant state of spores. Consequently, the ingestion activity of mononuclear phagocytic cells could be diminished. Accordingly, we found that a single-dose application of fixed *A. fumigatus* conidia did not lead to conidia uptake by conducting airway IE-DCs, but such interaction could occur in cases of a sensitized host and/or growing pathogens.

Although no IE-DC–conidia interactions were observed, we showed multiple contacts between IE-DCs and CD11b^+^ phagocytes in the conducting airway walls upon inflammatory response development and progression. We suggest that such interactions help to sustain the airway epithelial tissue integrity and prevent the damage that could occur due to massive neutrophil infiltration. Infiltrated tissue neutrophils were shown to form swarming clusters that could grow substantially due to neutrophil death and promote new neutrophil recruitment and subsequent inflammation progression (28, 36). However, the decrease in neutrophil number that we demonstrated at the late stage of single-dose *A. fumigatus* conidia inhalation indicated the absence of the inflammation progression due to some regulatory mechanisms activation. The involvement of DNGR-1-expressing cDC1s in the regulation of the fungus-induced neutrophil response was recently shown using a chronic candidiasis model (11). Upon regulation of lung inflammatory processes, cDC1s have been reported to mainly implement two biased strategies: apoptotic cell clearance and control of T cell-mediated responses by presenting or cross-presenting apoptotic cell antigens (37, 38). The IE-DC–CD11b^+^ phagocyte contacts described in the present study may represent instances of efferocytosis of apoptotic neutrophils. In some contacts (see an example in Figure 5B), the morphology of the proximate to CD11b^+^ phagocyte IE-DC region resemble the phagocytic cup. Thus, the removal of apoptotic neutrophils from swarming clusters that we supposedly observed in the present study evidently inhibited swarm amplification. Due to their low motility, IE-DCs are unlikely to migrate to the draining lymph nodes, but they can also be involved in the activation of T cells in the periphery (8). The production of IL-2 by CD103^+^ lung DCs was recently shown to play a fundamental role in the suppression of the Th17/IL-17 proinflammatory response to *A. fumigatus* (9). Although in the present study we did not check the cytokine production, IE-DCs can potentially be implicated in IL-17 downregulation. As IL-17 also contributes to neutrophil recruitment (39), such downregulation can suppress neutrophil infiltration and inflammation resolution, however, this phenomenon should be investigated in more detail in the future.

Conducting airway IE-DCs were characterized for the first time almost 30 years ago as MHCII-bearing cells with the “classical dendritic cell morphology” that formed the dense network in the airways (40). Later investigations confirmed that at steady state, these cells mostly express CD11c (8). Here, we show that upon *A. fumigatus* conidia-induced inflammation development, some CD11c^−^APCs (MHCII^+^CD11c^−^ cells with the morphology of IE-DCs) infiltrate the conducting airway wall. These cells are unlikely to be related to DCs with low surface expression of CD11c (41) because the usage of CD11c-EYFP mice in our study indicated the absence of CD11c expression at the transcript level. As far as significant changes in these cell numbers and in the numbers of CD11c^+^ cells in the conducting airway wall are observed during the late phase of single-dose conidia-induced inflammation, we can assume their contribution to the resolution of inflammation. It was demonstrated that neutrophil depletion induced enhanced CD11b^hi^CD11c^+^ DC recruitment to the lung (42). Here, we examined the effect of neutropenia on IE-DCs. In contrast to immunocompetent mice, no alteration of intraepithelial IE-DC numbers during the late phase of inflammation was detected in neutropenic mice. Recently infiltration of CX3CR1^+^ monocytes to the peritoneal wall in response to the swarming neutrophils (but not in case of neutrophil depletion) was demonstrated in the inflammatory responses to sterile tissue damage (43). As airway mucosal IE-DCs were also characterized as CX3CR1^+^ cells (16), these cells can also contribute to limit the growth of the neutrophil swarm. Thus, the observation that in the absence of neutrophils, there was no conducting airway wall IE-DC number elevation provides evidence of the contribution of these cells to neutrophil swarming prevention and dead neutrophil elimination (43, 44). In addition, the difference in the kinetics of conducting airway wall IE-DCs between immunocompetent and neutropenic mice, which we found here, contributes to the understanding of the susceptibility of immunocompromised patients to fungal infection.

**Figure 6:**
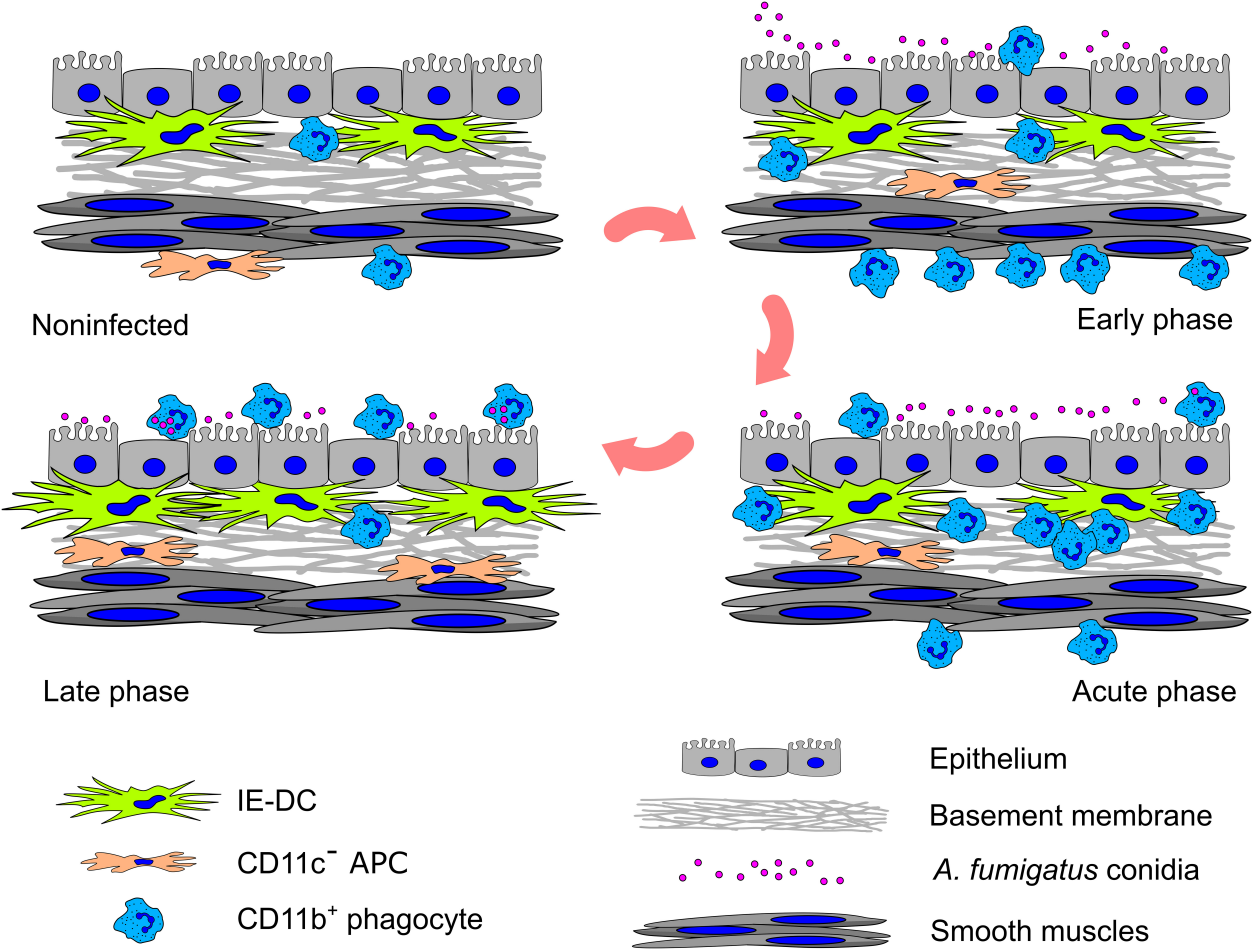
Graphical summary of the cell–pathogen and cell–cell interactions in the conducting airway mucosa during *A. fumigatus* conidia-induced inflammation. A small amount of CD11b^+^ phagocytes (mainly Ly6G^+^ neutrophils) presents in the conducting airway mucosa of uninfected mice. During the inflammatory response, these cells infiltrate the airways: in the early phase CD11b^+^ phagocytes accumulate in the submucosa in close proximity to smooth muscles; in the acute phase CD11b^+^ phagocytes penetrate the airway wall, and then pass through the epithelium and reach the airway lumen, where some of them interact with *A. fumigatus* conidia. Entering the conducting airway wall phagocytes encounter residing there IE-DCs. By the direct contact with CD11b^+^ phagocytes, IE-DCs contribute to regulating phagocytic response. The amount of IE-DCs increased in the late phase of inflammation. CD11c^−^ APCs also migrate from the submucosa to the conducting airway wall during the inflammation. IE-DCs and CD11c^−^ APCs form a network of intraepithelial APCs.

In summary, based on a detailed analysis of the spatiotemporal aspects of the local airborne pathogen-induced innate immune response, we observed that resident conducting airway IE-DCs are unlikely to directly contact pathogens but interact with phagocytes, invading the tissues in close proximity to pathogens. In response to aspirated airborne pathogens, phagocytes migrate from the submucosa to the luminal side of conducting airway epithelium. Entering the conducting airway wall phagocytes encounter resident IE-DCs (Figure 6). Since their discovery, IE-DCs have been named as “gatekeepers” or “sentinels” that monitor the environment and sense incoming antigens (45, 46). Here, we provide an anatomical basis for the contribution of IE-DCs to controlling the intrinsic danger of excessive phagocyte-mediated immune responses. Our findings reflect the interplay between antifungal and homeostatic immunity at the site of inflammation.

## Data Availability Statement

The datasets generated for this study are available on request to the corresponding author.

## Ethics Statement

The animal study was reviewed and approved by Institutional Animal Care and Use Committee at Shemyakin and Ovchinnikov Institute of Bioorganic Chemistry Russian Academy of Sciences.

## Author Contributions

MS contributed to the conception and design of the work and wrote the first draft of the manuscript. AOB, EB, TG, NT, AM, IO, and MS performed experiments and analyzed data. NT and MS performed the statistical analysis. EB, ES, and MS performed the animal experiments. TG, AB, VG, AS, and VB revised the manuscript. VB and MS supervised the project. All authors read and approved the submitted version.

### Conflict of Interest

The authors declare that the research was conducted in the absence of any commercial or financial relationships that could be construed as a potential conflict of interest.

## References

[1] Van De Veerdonk FL Gresnigt MS Romani L Netea MG Latgé JP. *Aspergillus fumigatus* morphology and dynamic host interactions. Nat Rev Microbiol. (2017) 15:661–74. 10.1038/nrmicro.2017.90 28919635

[2] Fisher MC Hawkins NJ Sanglard D Gurr SJ. Worldwide emergence of resistance to antifungal drugs challenges human health and food security. Science. (2018) 360:739–42. 10.1126/science.aap7999 29773744

[3] Bozza S Perruccio K Montagnoli C Gaziano R Bellocchio S Burchielli E. A dendritic cell vaccine against invasive aspergillosis in allogeneic hematopoietic transplantation. Blood. (2003) 102:3807–14. 10.1182/blood-2003-03-0748 12791648

[4] Espinosa V Rivera A. First line of defense: innate cell-mediated control of pulmonary Aspergillosis. Front Microbiol. (2016) 7:272. 10.3389/fmicb.2016.00272 26973640 PMC4776213

[5] Veres TZ Voedisch S Spies E Tschernig T Braun A. Spatiotemporal and functional behavior of airway dendritic cells visualized by two-photon microscopy. Am J Pathol. (2011) 179:603–9. 10.1016/j.ajpath.2011.04.039 21708113 PMC3157232

[6] Shevchenko MA Bolkhovitina EL Servuli EA Sapozhnikov AM. Elimination of *Aspergillus fumigatus* conidia from the airways of mice with allergic airway inflammation. Respir Res. (2013) 14:78. 10.1186/1465-9921-14-78 23890251 PMC3735401

[7] Jakubzick C Helft J Kaplan TJ Randolph GJ. Optimization of methods to study pulmonary dendritic cell migration reveals distinct capacities of DC subsets to acquire soluble versus particulate antigen. J Immunol Methods. (2008) 337:121–31. 10.1164/ajrccm-conference.2009.179.1_MeetingAbstracts.A4292 18662693 PMC3537501

[8] Veres TZ Kopcsányi T van Panhuys N Gerner MY Liu Z Rantakari P. Allergen-induced CD4 + T cell cytokine production within airway mucosal dendritic cell–T cell clusters drives the local recruitment of Myeloid effector cells. J Immunol. (2017) 198:895–907 10.4049/jimmunol.1601448 27903737 PMC5225021

[9] Zelante T Wong AYW Ping TJ Chen J Sumatoh HR Viganò E. CD103^+^ Dendritic Cells Control Th17 Cell Function in the Lung. Cell Rep. (2015) 12:1789–801. 10.1016/j.celrep.2015.08.030 26365185

[10] Vroman H Hendriks RW Kool M Dendritic cell subsets in asthma: impaired Tolerance or exaggerated inflammation? Front Immunol. (2017) 8:941 10.3389/fimmu.2017.00941 28848549 PMC5552666

[11] Del Fresno C Saz-Leal P Enamorado M Wculek SK Martínez-Cano S Blanco-Menéndez N. DNGR-1 in dendritic cells limits tissue damage by dampening neutrophil recruitment. Science. (2018) 362:351–6. 10.1126/science.aan8423 30337411

[12] Guilliams M Dutertre CA Scott CL McGovern N Sichien D Chakarov S. Unsupervised high-dimensional analysis aligns dendritic cells across tissues and species. Immunity. (2016) 45:669–84. 10.1016/j.immuni.2016.08.015 27637149 PMC5040826

[13] Hoffmann FM Berger JL Lingel I Laumonnier Y Lewkowich IP Schmudde I. Distribution and interaction of murine pulmonary phagocytes in the naive and allergic lung. Front Immunol. (2018) 9:1046. 10.3389/fimmu.2018.01046 29868009 PMC5964136

[14] Conejero L Khouili SC Martínez-Cano S Izquierdo HM Brandi P Sancho D. Lung CD103+ dendritic cells restrain allergic airway inflammation through IL-12 production. JCI Insight. (2017) 2:e90420. 10.1172/jci.insight.90420 28515363 PMC5436540

[15] Veres TZ Rochlitzer S Shevchenko M Fuchs B Prenzler F Nassenstein C. Spatial interactions between dendritic cells and sensory nerves in allergic airway inflammation. Am J Respir Cell Mol Biol. (2007) 37:553–61. 10.1165/rcmb.2007-0087OC 17600312

[16] Veres TZ Voedisch S Spies E Valtonen J Prenzler F Braun A. Aeroallergen challenge promotes dendritic cell proliferation in the airways. J Immunol. (2013) 190:897–903. 10.4049/jimmunol.1200220 23267021

[17] Moalli F Doni A Deban L Zelante T Zagarella S Bottazzi B Role of complement and Fcγ receptors in the protective activity of the long pentraxin PTX3 against *Aspergillus fumigatus*. Blood. (2010) 116:5170–80. 10.1182/blood-2009-12-258376 20829368

[18] Ramirez-Ortiz ZG Means TK The role of dendritic cells in the innate recognition of pathogenic fungi (A. fumigatus, C. neoformans and C. albicans). Virulence. (2012) 15:635–46. 10.4161/viru.22295 PMC3545945 23076328

[19] Lindquist RL Shakhar G Dudziak D Wardemann H Eisenreich T Dustin ML. Visualizing dendritic cell networks *in vivo*. Nat Immunol. (2004) 5:1243–50. 10.1038/ni1139 15543150

[20] Lother J Breitschopf T Krappmann S Morton CO Bouzani M Kurzai O. Human dendritic cell subsets display distinct interactions with the pathogenic mould *Aspergillus fumigatus*. Int J Med Microbiol. (2014) 304:1160–8. 10.1016/j.ijmm.2014.08.009 25200858

[21] Rao GVS Tinkle S Weissman DN Antonini JM Kashon ML Salmen R. Efficacy of a technique for exposing the mouse lung to particles aspirated from the pharynx. J Toxicol Environ Heal A. (2003) 66:1441–52. 10.1080/15287390306417 12857634

[22] Shevchenko MA Bogorodskiy AO Troyanova NI Servuli EA Bolkhovitina EL Büldt G. *Aspergillus fumigatus* infection-induced neutrophil recruitment and location in the conducting airway of immunocompetent, neutropenic, and immunosuppressed mice. J Immunol Res. (2018) 2018:5379085. 10.1155/2018/5379085 29577051 PMC5822902

[23] Scott GD Blum ED Fryer AD Jacoby DB. Tissue optical clearing, three-dimensional imaging, and computer morphometry in whole mouse lungs and human airways. Am J Respir Cell Mol Biol. (2014) 51:43–55. 10.1165/rcmb.2013-0284OC 24471696 PMC4091855

[24] Oremland M Michels KR Bettina AM Lawrence C Mehrad B Laubenbacher R. A computational model of invasive aspergillosis in the lung and the role of iron. BMC Syst Biol. (2016) 10:34. 10.1186/s12918-016-0275-2 27098278 PMC4839115

[25] Aimanianda V Bayry J Bozza S Kniemeyer O Perruccio K Elluru SR. Surface hydrophobin prevents immune recognition of airborne fungal spores. Nature. (2009) 460:1117–21. 10.1038/nature08264 19713928

[26] Akoumianaki T Kyrmizi I Valsecchi I Gresnigt MS Samonis G Drakos E. Aspergillus cell wall melanin blocks LC3-associated phagocytosis to promote pathogenicity. Cell Host Microbe. (2016) 19:79–90. 10.1016/j.chom.2015.12.002 26749442

[27] Lukácsi S Nagy-Baló Z Erdei A Sándor N Bajtay Z. The role of CR3 (CD11b/CD18) and CR4 (CD11c/CD18) in complement-mediated phagocytosis and podosome formation by human phagocytes. Immunol Lett. (2017) 189:64–72. 10.1016/j.molimm.2017.06.093 28554712

[28] Lämmermann T. In the eye of the neutrophil swarm-navigation signals that bring neutrophils together in inflamed and infected tissues. J Leukoc Biol. (2016) 100:55–63. 10.1189/jlb.1MR0915-403 26416718

[29] Latgé JP. *Aspergillus fumigatus* and Aspergillosis. Clin Microbiol Rev. (1999) 12:310–50. 10.1128/CMR.12.2.310 10194462 PMC88920

[30] Graham IL Gresham HD Brown EJ. An immobile subset of plasma membrane CD11b/CD18 (Mac-1) is involved in phagocytosis of targets recognized by multiple receptors. J Immunol. (1989) 142:2352–8. 2538507

[31] Teschner D Cholaszczynska A Ries F Beckert H Theobald M Grabbe S CD11b regulates fungal outgrowth but not neutrophil recruitment in a mouse model of invasive pulmonary aspergillosis. Front Immunol. (2019) 10:123 10.3389/fimmu.2019.00123 30778357 PMC6369709

[32] Kalleda N Amich J Arslan B Poreddy S Mattenheimer K Mokhtari Z. Dynamic immune cell recruitment after murine pulmonary *Aspergillus fumigatus* infection under different immunosuppressive regimens. Front Microbiol. (2016) 7:1107. 10.3389/fmicb.2016.01107 27468286 PMC4942482

[33] Ziegler S Weiss E Schmitt AL Schlegel J Burgert A Terpitz U. CD56 is a pathogen recognition receptor on human natural killer cells. Sci Rep. (2017) 7:6138. 10.1038/s41598-017-06238-4 28733594 PMC5522490

[34] Renkawitz J Kopf A Stopp J de Vries I Driscoll MK Merrin J. Nuclear positioning facilitates amoeboid migration along the path of least resistance. Nature. (2019) 568:546–50. 10.1038/s41586-019-1087-5 30944468 PMC7217284

[35] Bose O Baluk P Looney MR Cheng LE McDonald DM Caughey GH. Mast cells present protrusions into blood vessels upon tracheal allergen challenge in mice. PLoS ONE. (2015) 10:e0118513. 10.1371/journal.pone.0118513 25789765 PMC4366375

[36] Lämmermann T Afonso PV Angermann BR Wang JM Kastenmüller W Parent CA. Neutrophil swarms require LTB4 and integrins at sites of cell death *in vivo*. Nature. (2013) 498:371–5. 10.1038/nature12175 23708969 PMC3879961

[37] Desch AN Randolph GJ Murphy K Gautier EL Kedl RM Lahoud MH. CD103 + pulmonary dendritic cells preferentially acquire and present apoptotic cell-associated antigen. J Exp Med. (2011) 208:1789–97. 10.1084/jem.20110538 21859845 PMC3171085

[38] Espinosa-Cueto P Magallanes-Puebla A Castellanos C Mancilla R. Dendritic cells that phagocytose apoptotic macrophages loaded with mycobacterial antigens activate CD8 T cells via cross-presentation. PLoS ONE. (2017) 12:e0182126. 10.1371/journal.pone.0182126 28767693 PMC5540487

[39] Rosales C Neutrophil: A cell with many roles in inflammation or several cell types? Front Physiol. (2018) 9:113 10.3389/fphys.2018.00113 29515456 PMC5826082

[40] Schon-Hegrad MA Oliver J McMenamin PG Holt PG. Studies on the density, distribution, and surface phenotype of intraepithelial class II major histocompatability complex antigen (Ia)-bearing dendritic cells (DC) in the conducting airways. J Exp Med. (1991) 173:1345–56. 10.1084/jem.173.6.1345 2033368 PMC2190835

[41] Osorio F Tavernier SJ Hoffmann E Saeys Y Martens L Vetters J. The unfolded-protein-response sensor IRE-1α regulates the function of CD8α + dendritic cells. Nat Immunol. (2014) 15:248–57. 10.1038/ni.2808 24441789

[42] Park SJ Burdick MD Brix WK Stoler MH Askew DS Strieter RM. Neutropenia enhances lung dendritic cell recruitment in response to aspergillus via a cytokine-to-chemokine amplification loop. J Immunol. (2010) 185:6190–7. 10.4049/jimmunol.1002064 20926800 PMC3032263

[43] Uderhardt S Martins AJ Tsang JS Lämmermann T Germain RN. Resident macrophages cloak tissue microlesions to prevent neutrophil-driven inflammatory damage. Cell. (2019) 177:541–555.e17. 10.1016/j.cell.2019.02.028 30955887 PMC6474841

[44] Grabowska J Lopez-Venegas MA Affandi AJ Den Haan JMM. CD169+ macrophages capture and dendritic cells instruct: The interplay of the gatekeeper and the general of the immune system. Front Immunol. (2018) 9:2472. 10.3389/fimmu.2018.02472 30416504 PMC6212557

[45] Holt PG Stumbles PA. Regulation of immunologic homeostasis in peripheral tissues by dendritic cells: the respiratory tract as a paradigm. J Allergy Clin Immunol. (2000) 105:421–9. 10.1067/mai.2000.105010 10719288

[46] Lambrecht BN Prins JB Hoogsteden HC. Lung dendritic cells and host immunity to infection. Eur Respir J. (2001) 18:692–704. 11716176

