# Supplementary material for: Murine Intraepithelial Dendritic Cells Interact With Phagocytic Cells During Aspergillus fumigatus-Induced Inflammation
Source: Front Immunol. 2020 Feb 25;11:298. doi: 10.3389/fimmu.2020.00298 (PMC7053491; doi:10.3389/fimmu.2020.00298)
Supplement: Supplementary file 1 [file Data_Sheet_1.pdf]

# *Supplementary Material*

## 1 Supplementary Figures

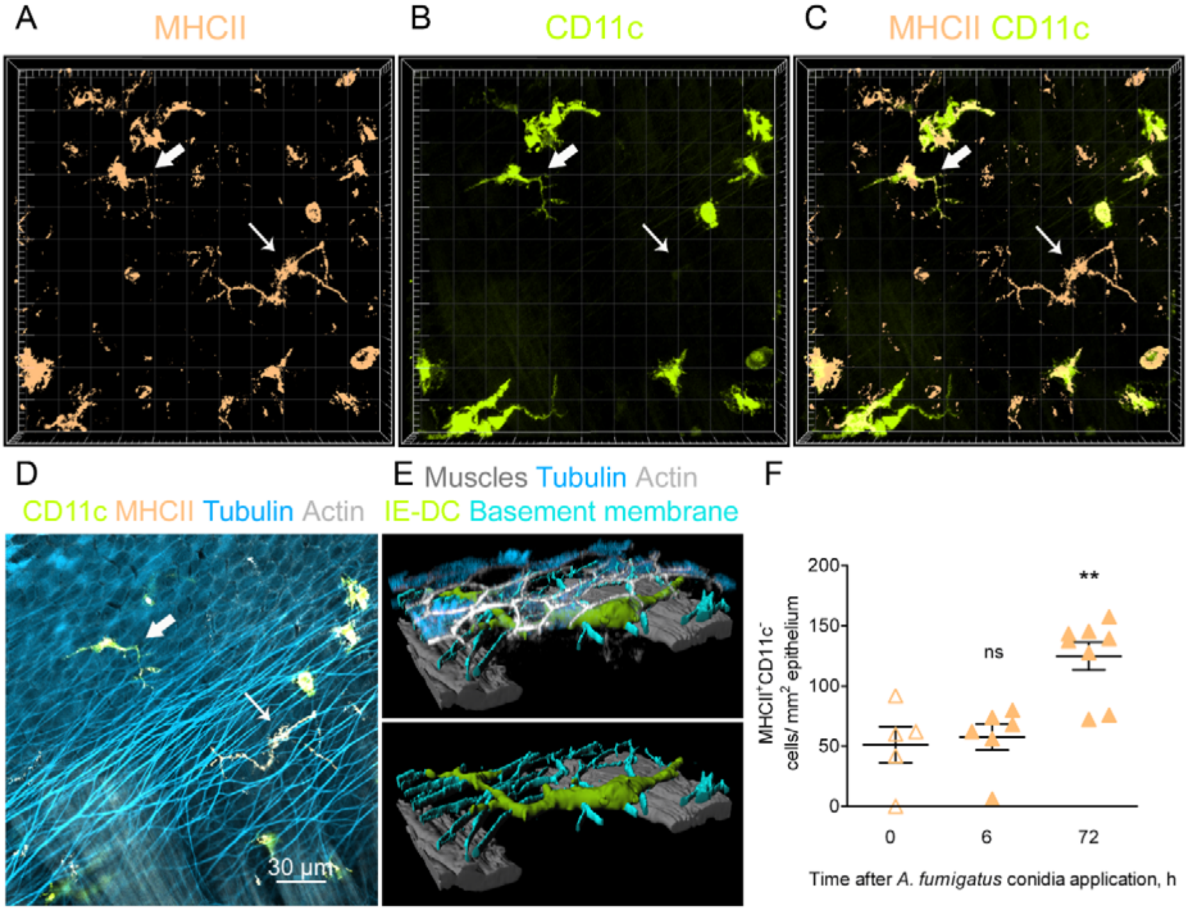


**Supplementary Figure 1.** CD11c$^+$ and CD11c$^-$ intraepithelial APCs of the conducting airways of *A. fumigatus* conidia-infected mice. Representative three-dimensional image of a region of the conducting airway mucosa of mice 6 hours after conidia application. (**A**) MHCII$^+$ intraepithelial APCs (orange). (**B**) CD11c$^+$ cells (green). (**C**) Merged. Grid spacing, 20 µm. (**D**) Image of the same region, showing MHCII$^+$ CD11$^-$ cell and IE-DC and actin fibers (grey) and tubulin (light blue). Typical intraepithelial APCs are indicated with arrows: IE-DC with bold arrow, MHCII$^+$ CD11c$^-$ cell with fine arrow. Image is presented as XY projection of a single layer, indicating the position of intraepithelial APCs in relation to the basement membrane (tubulin, light blue). Scale bar, 30 µm. (**E**) Enlarged image of IE-DC that is indicated with bold arrow in (**D**). IE-DC (green), basement membrane (cyan) and smooth muscles (dark grey) are represented via surface rendering (upper and lower images). Actin-reach epithelial cell contacts that are visualized by staining with Phalloidin (grey) and cilia that are visualized by staining with Tubulin (light blue) are represented via volume rendering (upper image). (**F**)The numbers of MHCII$^+$CD11c$^-$ cell in the airway wall of mice at 0, 6 and 72 hours after conidia application (n = 5–10 mice per time point; each data point is an average of n = 2–4 tiles per mouse). The data are shown as the median and IQR. Statistical analysis was performed using the Mann–

Whitney U test. Significant differences between the indicated time point and the 0 hour time point are indicated: **: $p \leq 0.01$; ns – no significant difference.

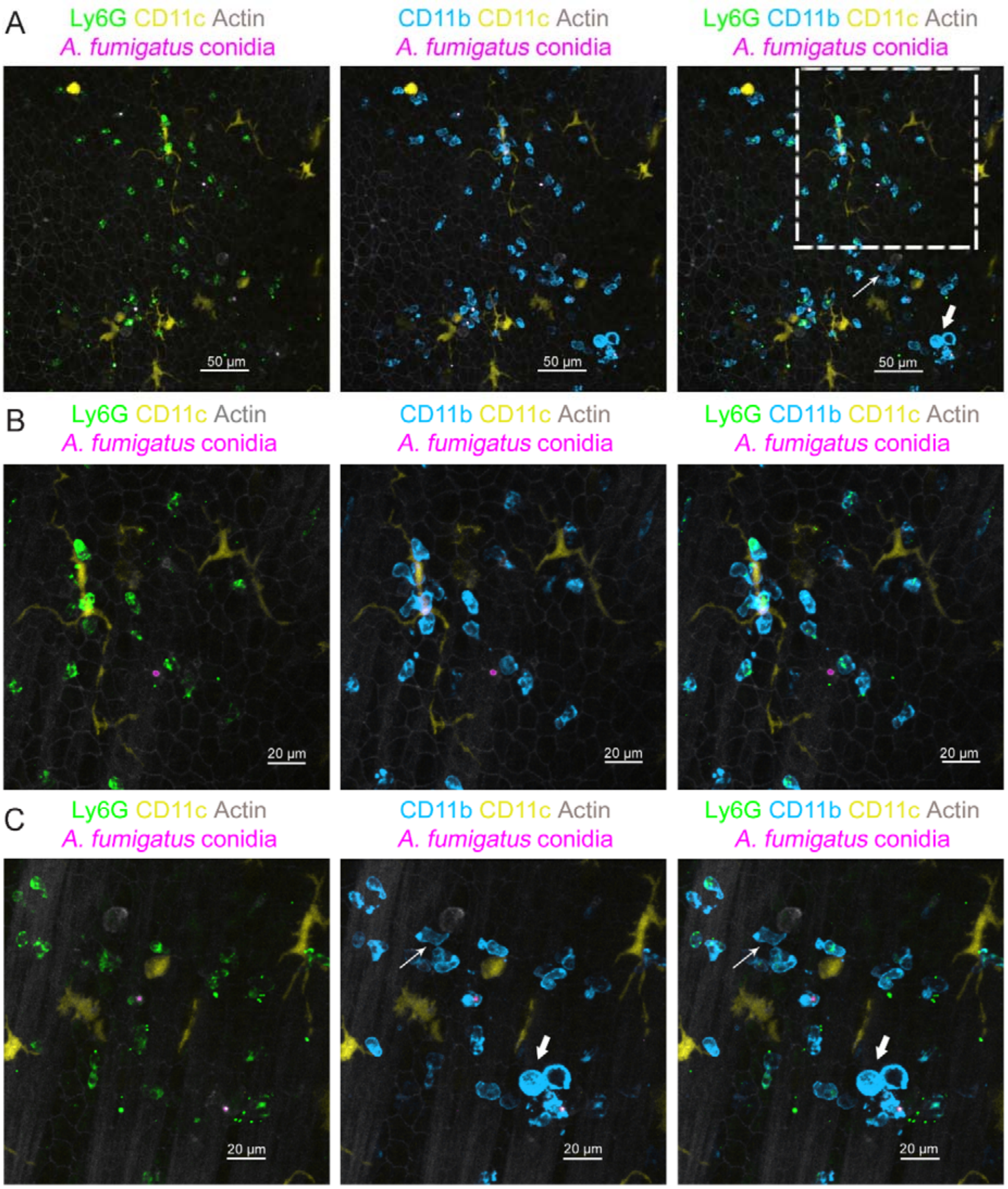


**Supplementary Figure 2.** Identification of Ly6G$^+$ neutrophils among CD11b$^+$ phagocytes. **(A)** Representative three-dimensional image of a region of the mouse conducting airway mucosa 24 hours after conidia application. Ly6G$^+$ neutrophils (green), CD11b$^+$ phagocytes (light blue), CD11c$^+$ cells, including IE-DCs (yellow), *A. fumigatus* conidia (magenta) and actin fibers (grey) are represented via volume rendering. Scale bar, 50 µm. **(B)** Higher magnification image

of the region indicated in **(A)** showing Ly6G$^+$ neutrophils expressing CD11b and interacting with IE-DCs. Scale bar, 20 µm. **(C)** Higher magnification image of Ly6G$^-$CD11b$^+$ cells that are indicated with fine and bold arrows in **(A)**. Scale bar, 20 µm.

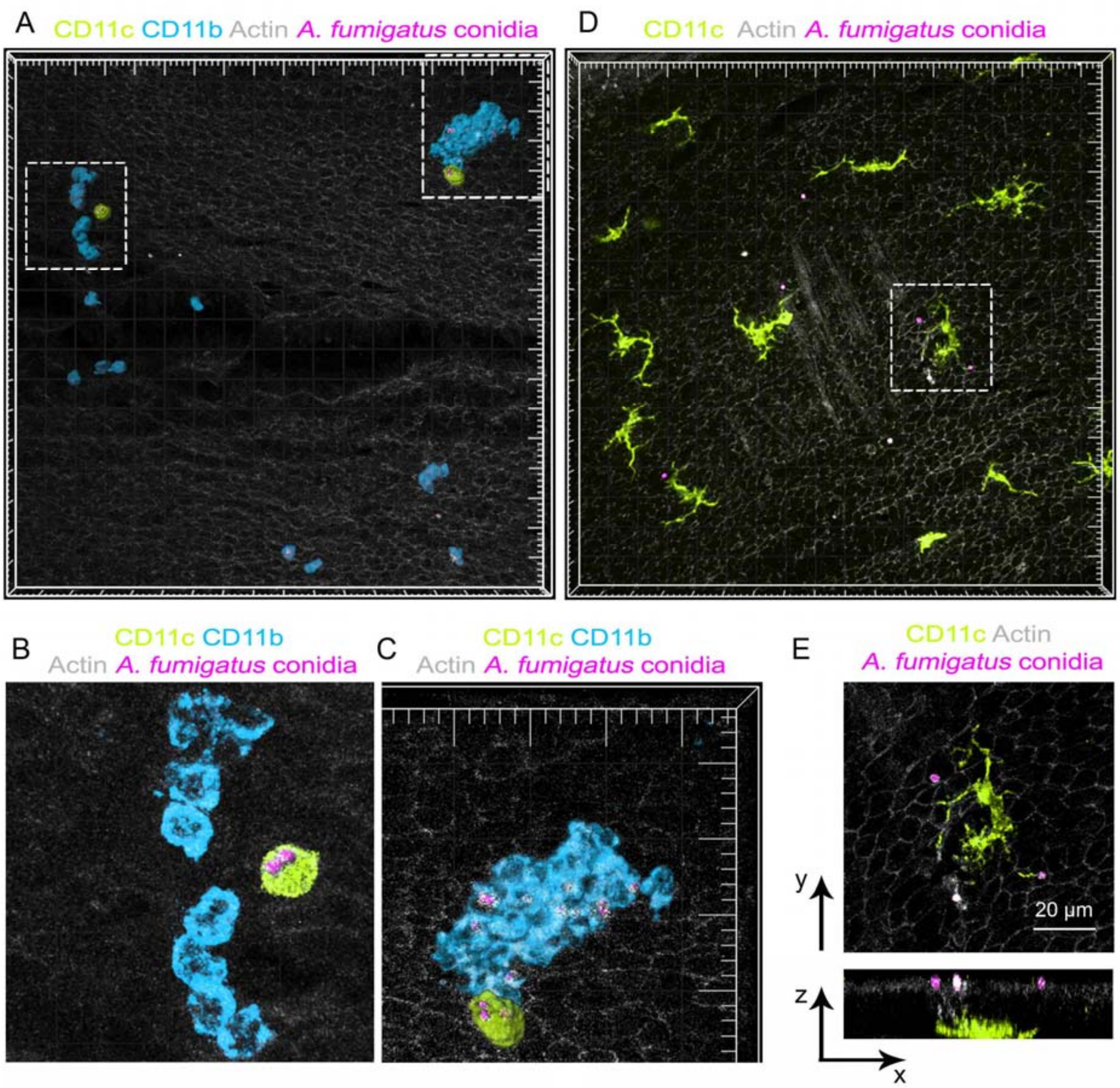


**Supplementary Figure 3.** Internalization of *A. fumigatus* conidia by conducting airway mucosal CD11c$^+$ cells and CD11b$^+$ phagocytes. **(A)** Representative images of *A. fumigatus* conidia (magenta) internalization events by CD11c$^+$ cells (green) or CD11b$^+$ phagocytes (light blue) on the luminal side of the conducting airway epithelium (grey) at 24 hours after conidia application. Grid spacing, 20 µm. **(B), (C)** Enlarged regions that are boxed in **(A)** showing internalization of conidia (magenta) by CD11c$^+$ cells (green) or by CD11b$^+$ phagocytes (light blue). Cells and conidia are represented via surface rendering, and the epithelium is represented via volume rendering. Grid spacing, 20 µm. **(D)** IE-DCs (green) that are located below the epithelium (grey) and above the smooth muscles (grey). Grid spacing, 20 µm. **(E)** Precise location of IE-DCs (green) below the epithelium (grey) and conidia (magenta) on the luminal side of the epithelium represented as x-y (upper image) and x-z (lower image) projections. Scale bar, 20 µm.
